# Loss of protection with insecticide-treated nets against pyrethroid-resistant *Culex quinquefasciatus *mosquitoes once nets become holed: an experimental hut study

**DOI:** 10.1186/1756-3305-1-17

**Published:** 2008-06-18

**Authors:** SR Irish, R N'Guessan, PM Boko, C Metonnou, A Odjo, M Akogbeto, M Rowland

**Affiliations:** 1 London School of Hygiene and Tropical Medicine, Keppel Street, WC1E 7HT, London, UK; 2 Le Centre de Recherche Entomologique de Cotonou (CREC), 06 BP 2604, Cotonou, Bénin

## Abstract

**Background:**

An important advantage of pyrethroid-treated nets over untreated nets is that once nets become worn or holed a pyrethroid treatment will normally restore protection. The capacity of pyrethroids to kill or irritate any mosquito that comes into contact with the net and prevent penetration of holes or feeding through the sides are the main reasons why treated nets continue to provide protection despite their condition deteriorating over time. Pyrethroid resistance is a growing problem among Anopheline and Culicine mosquitoes in many parts of Africa. When mosquitoes become resistant the capacity of treated nets to provide protection might be diminished, particularly when holed. An experimental hut trial against pyrethroid-resistant *Culex quinquefasciatus *was therefore undertaken in southern Benin using a series of intact and holed nets, both untreated and treated, to assess any loss of protection as nets deteriorate with use and time.

**Results:**

There was loss of protection when untreated nets became holed; the proportion of mosquitoes blood feeding increased from 36.2% when nets were intact to between 59.7% and 68.5% when nets were holed to differing extents. The proportion of mosquitoes blood feeding when treated nets were intact was 29.4% which increased to 43.6–57.4% when nets were holed. The greater the number of holes the greater the loss of protection regardless of whether nets were untreated or treated. Mosquito mortality in huts with untreated nets was 12.9–13.6%; treatment induced mortality was less than 12%. The exiting rate of mosquitoes into the verandas was higher in huts with intact nets.

**Conclusion:**

As nets deteriorate with use and become increasingly holed the capacity of pyrethroid treatments to restore protection is greatly diminished against resistant *Culex quinquefasciatus *mosquitoes.

## Background

Pyrethroid-treated nets are an important method of preventing malaria mortality and morbidity in sub-Saharan Africa. Untreated nets are able to provide some protection against infective mosquitoes but once they become holed, which they do with time, protection is lost [1]. Pyrethroid insecticides have the capacity to induce excito-repellency in mosquitoes that come into contact with treated nets; this property will restore efficacy even when nets become holed [1,2]. For this reason, when any new pyrethroid or long-lasting insecticidal net is submitted to the WHO Pesticide Evaluation Scheme for evaluation or approval, the testing is done on holed rather than intact nets [3].

The nuisance mosquito and filariasis vector *Culex quinquefasciatus *is resistant to pyrethroids across West and East Africa [4,5]. Field trials of ITNs conducted in experimental huts record low rates of mortality against this species [4,6]. The malaria vector *Anopheles gambiae sensu stricto *has also developed resistance to pyrethroids in West and East Africa [7,8] and, as in *Culex*, the resistance can be caused by *kdr*, oxidases, esterases or a combination of mechanisms [7,9,10]. The resistance conferred by *kdr *does not appear to be particularly strong in *An. gambiae *of the S molecular form. In the presence of ITNs this biotype shows high rates of mortality and low rates of blood feeding even when the nets have many holes [11]. Recently however, a form of pyrethroid resistance has been found in *An. gambiae *of the M molecular form (also associated with the *kdr *mechanism) that seems to be highly protective [12,13]. Resistant mosquitoes of this M biotype readily penetrate holed ITNs and feed more successfully than mosquitoes of the S molecular form [11,12]. The failure of ITNs once holed to protect against certain types of pyrethroid-resistant *Culex *and *Anopheles *needs further study since it is the relationship between pyrethroid dosage on nets, the level of resistance in the mosquito, and number or area of holes that determines whether an ITN is protective to users.

The WHO guidelines on the evaluation of ITNs requires six holes measuring 4 cm by 4 cm to be cut into the side and end panels to simulate a worn or holed net [3]. Previously ITNs were evaluated in West Africa with far greater numbers of holes per net (80 holes) but of smaller size (2 cm by 2 cm) [11,12]. The effect that differences in the number and size of holes and number may have on efficacy of treated nets has not been determined before and is important if results of earlier investigations are to be compared with more recent ones. This paper describes an experimental hut trial conducted in southern Benin using untreated and insecticide-treated nets that were deliberately holed in order to explore the relationship between insecticide resistance, insecticide treatment and deterioration in the physical integrity of the material (number of holes) on the capacity of ITNs to protect against *Culex quinquefasciatus *mosquitoes.

## Materials and methods

### Study site and experimental huts

The study was carried out in six identical experimental huts sited on the shore of Lake Nokoué in Ladji, Cotonou, in southern Benin. The huts were built in the style of Darriet et al. [14] but were fixed upon concrete pillars 1.2 meters above the ground to keep the huts above water level during seasonal flooding. The walls were made of brick and plastered with cement, the roof made of corrugated iron and the ceiling of thick polyethylene sheeting. Four window slits on three walls of the hut allowed host-seeking mosquitoes to freely enter the room. A screened veranda trap projecting from the fourth wall captured any mosquito that attempted to exit via that route. Movement of mosquitoes between the room and the veranda was unimpeded.

### Mosquito net treatment

The nets were made of white, 100-denier polyester netting (SiamDutch Mosquito Netting Co., Bangkok, Thailand), measuring 2.11 m long, 1.63 m wide and 1.84 m high, and had a surface area of 17.2 m^2^. Some of the nets were untreated and others were treated with alphacypermethrin (Fendona SC 6%; BASF, Research Triangle Park, NC, USA) at a rate of 40 mg/m^2^. The following nets were tested:

1. Intact net (unholed) and untreated.

2. Intact net (unholed) and treated with 40 mg/m^2 ^alphacypermethrin.

3. Net with 80 evenly spaced holes, each hole measuring 2 × 2 cm, cut in the side and end panels, and untreated.

4. Net with 80 evenly spaced holes, each hole measuring 2 × 2 cm, cut in the side and end panels, and treated with 40 mg/m^2 ^alphacypermethrin.

5. Net with 6 holes, each hole measuring 4 × 4 cm, two on each side and one at each end, and untreated.

6. Net with 6 holes, each hole measuring 4 × 4 cm, two on each side and one at each end, treated with 40 mg/m^2 ^alphacypermethrin.

### Sleepers and mosquito collection

Preliminary collections were made in sleeper occupied huts over 12 nights to assess the relative attractiveness of huts and sleepers. The intervention trial was conducted over 36 nights from 6 August to 15 September 2007.

Six adult men were trained in the collection of mosquitoes. The men slept overnight in the huts and collected mosquitoes from the huts at 6:00 in the mornings. They were rotated between huts on successive nights to adjust for any differences in attractiveness between individual huts or sleepers. Treatments were assigned randomly to huts and were then rotated weekly between huts until each treatment had been evaluated in each hut. At the end of each week, the bedding and floors of the huts were thoroughly cleaned using detergent. Each morning, mosquitoes were collected from the floors, walls, and ceilings of rooms, verandas and nets using aspirators and torches. The collecting from each hut was repeated by a second collector to ensure complete removal of mosquitoes. The mosquitoes were identified to species and recorded as live or dead and blood-fed or unfed at the laboratory. Live mosquitoes were provided with 10% honey solution and held for 24 hours before recording delayed mortality. Male mosquitoes were not scored.

The entomological impact of each treatment was expressed relative to the controls (untreated nets) in terms of: deterrence (the proportional reduction in the number of mosquitoes entering the treated huts relative to control huts), induced exiting (the proportions of mosquitoes collected in the veranda traps of the treatment huts relative to the verandas of the control huts), blood feeding inhibition (the proportional reduction in blood-feeding rates in the treatment huts relative to controls), and mortality (the proportions of mosquitoes found dead in the huts at the time of collection [immediate mortality] and after a 24 h holding period).

WHO cone bioassays were performed using *Anopheles gambiae *Kisumu, a laboratory pyrethroid-susceptible strain, at the beginning, middle and end of the trial to monitor any change in insecticide activity.

### Data analysis

The attractiveness of huts or treatments to mosquitoes was examined using Kruskal-Wallis analysis of variance. Proportional data (blood-feeding, mortality and exiting rates) were analysed using logistic regression (Stata 8 software, Stata Co., College Station, TX, USA) after adjusting for variation between sleepers and huts.

### Ethical clearance

The study procedures were approved by the London School of Hygiene and Tropical Medicine and Benin national ethics committees. Written informed consent was obtained from the sleepers to participate in the study.

## Results

Over the course of the trial, 1265 *Culex quinquefasciatus *and 39 *Anopheles gambiae s.l*. females were caught in the huts. Only results for *Culex quinquefasciatus *are presented (Table 1). Bioassay mortality on all treated nets was 100% (n = 160) before the trial and 98% (n = 151) at the end of the trial; hence the integrity of the treatment was consistent throughout.

**Table 1:** Blood-feeding, mortality, and exophily of *Culex *mosquitoes entering experimental huts*

**Net condition**	**Total number collected**	**% Blood-feeding (CI)**	**Blood-feeding inhibition %**	**% Entering the veranda trap (CI)**	**Overall mortality % (CI)**	**Immediate mortality % (CI)**
0 holes untreated	**177**	**36.2^ac ^**(29.4–43.5)	**-**	**44.6^a ^**(37.5–52.0)	**13.6^a ^**(9.3–19.4)	**5.6^ab ^**(3.1–10.2)
6 holes untreated	**263**	**59.7^bd ^**(53.7–65.5)	**-**	**28.5^bc ^**(23.4–34.3)	**12.9^a ^**(9.4–17.5)	**8.0^abc ^**(5.3–11.9)
80 holes untreated	**248**	**68.5^b ^**(62.5–74.0)	**-**	**26.6^bc ^**(21.5–32.5)	**12.9^a ^**(9.3–17.7)	**4.4^a ^**(2.5–7.8)
0 holes alphacypermethrin- treated	**187**	**29.4^a ^**(23.3–36.3)	**18.8**	**46.5^a ^**(39.5–53.7)	**21.9^bc ^**(16.6–28.4)	**13.4^cd ^**(9.2–19.0)
6 holes alphacypermethrin- treated	**202**	**43.6^c ^**(36.9–50.5)	**27.0**	**33.7^b ^**(27.5–40.5)	**23.3^bc ^**(18.0–29.6)	**15.3^d ^**(11.0–21.0)
80 holes alphacypermethrin- treated	**188**	**57.4^d ^**(50.3–64.3)	**16.2**	**24.5^c ^**(18.9–31.1)	**18.6^ac ^**(13.7–24.8)	**9.6^bcd ^**(6.1–14.7)

*The values in each column are significantly different if they do not share the same superscript letter.

No significant difference was recorded between the 6 treatments in the overall number of mosquitoes collected per treatment (*P *= 0.26).

There was an association between the proportion of *Culex quinquefasciatus *blood-feeding and the number of holes in the net (Table 1). For untreated nets there was a 1.64 increase in the proportion of mosquitoes blood-feeding through nets with 6 holes and a 1.89 increase in the proportion blood-feeding through nets with 80 holes compared to nets with no holes. For treated nets the proportional increase in feeding through nets with 6 or 80 holes compared to treated nets with no holes were 1.48 and 1.95 respectively. The difference in the proportion of mosquitoes that fed through treated nets with 6 or 80 holes was significant (P = 0.026). For untreated nets the difference in the proportion of mosquitoes that fed through nets with 6 and 80 holes was borderline significant (*P *= 0.064) and the trend was in the direction of more bloodfeeding with more holes. The level of blood feeding inhibition between treated and untreated nets (18.8%) when both were intact was not significant (P = 0.256). The level of blood-feeding inhibition between untreated and treated nets with the same configuration (i.e. same number of holes) was significant (6 holes, 27% inhibition, P = 0.001; 80 holes, 16.2% inhibition, P = 0.012) (Table 1). Thus the insecticide treatments provided only limited protection. With 29.4% of mosquitoes feeding through the treated net that had no holes, and with 43.6% (P = 0.013) and 57.4% (P = 0.001) feeding through the treated nets that had 6 and 80 holes respectively, the insecticide treatment was clearly failing to restore the efficacy of holed nets to that of unholed, intact nets.

Mortality associated with insecticide-treated nets was significantly higher than that for untreated nets of the equivalently holed or intact physical condition (for 80 holed nets the difference was not significant, P = 0.116). Mortality associated with the pyrethroid treatment did not differ significantly between intact or holed nets. After correcting for control mortality, insecticide-induced mortality was 6.5% for 80-hole nets, 11.9% for 6-hole nets and 9.6% for 0-hole nets.

Comparing treated nets with untreated nets of the same physical configuration, there was no evidence for insecticide-induced exiting of mosquitoes into the veranda traps. However, there were significant differences in exiting rates associated with the configuration of the nets. Exiting rates were significantly higher from huts containing intact nets than from huts containing holed nets.

## Discussion

*Culex quinquefasciatus *is an urban vector of lymphatic filariasis in East Africa and Asia and an important nuisance mosquito throughout its geographic range. It breeds in sites rich in organic matter and adults can reach very high densities. Biting times for *Culex quinquefasciatus *vary throughout its distribution; in West Africa, the peak biting time is between 22:00 and 2:00 am [15]. Often, the biting of *Culex *mosquitoes is a motivating factor for people to acquire and use bed nets, which has obvious repercussions for malaria control where *Culex *and *Anopheles *are sympatric [1].

In our experimental hut trial we examined the effect of mosquito nets either with or without holes, and with or without insecticide, on the blood-feeding rates of *Culex quinquefasciatus*. A high proportion of *Culex *was able to blood-feed even when nets were intact and insecticide-treated. Some of the mosquitoes may have blood-fed elsewhere and entered the huts in search of a refuge. Generally few *Culex *are found resting inside when huts are unoccupied. Hence the majority of mosquitoes in the current trial presumably entered the huts in search of blood source and succeeded in feeding either through the sides of the net or after penetrating the holes.

There was an association between the proportion of mosquitoes that blood-fed and the number of holes in the nets; this was significant for treated nets and showed a similar trend in untreated nets. The total area of holes was 320 cm^2 ^in nets with 80 holes and 96 cm^2 ^in nets with 6 holes. The difference in feeding success might be due to differences in the total area of holes rather than to the number of holes. At this point we are unable to distinguish between these possibilities.

The insecticide treatments provided little protection against blood feeding and induced negligible mortality among *Culex *mosquitoes. In a recent survey, pyrethroid-resistant *Culex quinquefasciatus *from southern Benin showed high survival (55%) when exposed to permethrin test papers, a *kdr *frequency of 63%, and elevated levels of oxidase and esterase enzymes relative to a laboratory standard susceptible strain [16]. Once a species develops high level resistance, the pyrethroid treatment becomes almost redundant and its capacity to restore holed nets to the status of an intact ITN is lost.

A trend of failing ITN against resistant mosquitoes might also be emerging against *An. gambiae s.s*. from southern Benin. In experimental hut trials conducted at the same site but at a time of year when the *An. gambiae *M biotype is abundant, survival rates (70%) and blood-feeding rates (82%) of this *kdr *bearing biotype remained high in the presence of holed ITNs [12]. This stands in contrast to *An. gambiae *S biotype from Côte d'Ivoire where ITN continue to prevent malaria and where *kdr *genotypes seem to confer little or no protection to mosquitoes that come into contact with ITNs [11,17].

Further investigations are needed to determine whether *Anopheles *behaviour around insecticide-treated nets is similar or different to that of *Culex *mosquitoes. Port and Boreham [18] evaluated the influence of holes in untreated bed nets on *Anopheles gambiae sensu stricto *in experimental huts in the Gambia. They found that blood-feeding increased with the size and number of holes. However, their studies were done using bed nets that were used by the local population, which meant that the sizes, shapes, and location of holes were not standardized, as is typically done in experimental hut studies.

It has been suggested that pyrethroid-treated nets might provide some protection against urban filariasis transmitted by *Culex quinquefasciatus *[1,19]. The present study indicates that any protection would be short lived once treated nets became holed. Alternative insecticides to pyrethroids to which *Culex *shows no resistance are required for ITNs in filariasis endemic areas or in places where *Culex *is a particular nuisance.

There was an inverse relationship between the rate of mosquito exiting from huts and the number of holes in the nets. This may reflect the higher rates of feeding success associated with holed nets, as unfed mosquitoes may leave experimental huts in greater numbers than fed mosquitoes to search for a blood-meal elsewhere [18]. The proportion of unfed mosquitoes in the veranda relative to the overall number of unfed mosquitoes in the hut was 48% in the presence of intact nets and 36% in the presence of holed nets, a trend that supports the idea that unfed mosquitoes leave to search elsewhere when thwarted by an intact net. The number of holes seemed more important than the presence of insecticide in inducing exiting behaviour, presumably because the high level of pyrethroid resistance in *Culex quinquefasciatus *is protective at this site [12,16]. Darriet et al. [20] compared the effects of intact and holed untreated nets against *An. gambiae s.s*. in Côte d'Ivoire; there was significantly higher exiting and lower blood-feeding when nets were intact than when they were holed.

## Conclusion

The capacity of pyrethroid treatment to render a net protective against pyrethroid-resistant *Culex quinquefasciatus *mosquitoes is diminished when nets become holed. The loss of protection is related to the number or area of holes. The capacity of alphacypermethrin-treated nets to kill pyrethroid-resistant *Culex quinquefasciatus *is limited. To restore protection will require a new generation of nets treated with a pyrethroid plus an insecticide to which *Culex *shows no resistance. It remains to be confirmed whether the association between the loss of protection and the number of holes also holds true for the pyrethroid-resistant *Anopheles gambiae *M biotype that occurs in Southern Benin [12].

## Competing interests

The authors declare that they have no competing interests.

## Authors' contributions

SI organized and managed the experimental hut trial and drafted the manuscript, RN participated in the design of the study and revised the manuscript, PB, CM, and AO conducted the bioassays and scored the results. MR conceived the study and revised the final manuscript. All authors read and approved the final manuscript.
